# Urticaria as initial finding of a patient with carcinoid tumor

**DOI:** 10.1186/s40413-015-0083-y

**Published:** 2015-12-10

**Authors:** Ivan Cherrez Ojeda, Juan Carlos Calderon, Karin Plaza, Emanuel Vanegas, Annia Cherrez, José Cano

**Affiliations:** Universidad de Especialidades Espíritu Santo, School of Medicine, Samborondón, Ecuador; Respiralab Research Group, Clínica Kennedy, Guayaquil, Ecuador; University of Heidelberg, School of Medicine, Heidelberg, Germany

**Keywords:** Urticaria, Quality of life, Carcinoid tumors, Kinin, Serotonin, Enterochromaffin cells, Antibodies

## Abstract

**Background:**

Typical carcinoid syndrome is characterized by flushing, abdominal pain and diarrhea and occurs in <10 % of carcinoid tumor patients. Very rarely, initial signs include skin manifestations. Our purpose is to highlight cutaneous manifestations in the diagnosis and assessment of a patient with atypical manifestation of type I gastric carcinoid tumor.

**Case presentation:**

A 50-year-old woman presented with anemia, chronic urticaria and angioedema. Urticaria was triggered principally by seafood and appeared in the first hour after. Urticaria Activity Score 7 was 24, and quality of life (CU-Q2oL) was 3.61. P. Laboratory findings showed anemia, diminished iron, ferritin, and vitamin B12, with increased gastrin and anti-parietal cell antibody levels. 15 gastric carcinoids 5 mm in diameter were observed in the greater curvature of the stomach during gastric endoscopy and confirmed by biopsy, suggesting that this patient had type I gastric carcinoids. Four additional tumors were found in the small intestine upon examination via video capsule. Endoscopic argon plasma therapy was performed. The patient experienced definitive improvement in quality of life and urticaria activity score.

**Conclusion:**

This patient, whose principal symptoms were anemia, urticaria and angioedema, was found to have atypical carcinoid syndrome, with tumors located in the stomach. Allergists, immunologists, internists and primary care physicians should consider the possibility of neuroendocrine malignancies, specifically type I carcinoid tumors, when evaluating patients with urticaria, and consider screening patients with chronic urticaria for elevated anti-parietal cell antibody levels.

## Background

Carcinoid tumors are rare, slow-growing neuroendocrine tumors arising from the enterochromaffin cells. They are most commonly found in the gastrointestinal tract (64 %) [1], although they can involve any organ. These tumors can secrete biogenic amines and peptides, such as ACTH, 5-HTP and serotonin [2]. Classical carcinoid syndrome occurs in <10 % of patients with carcinoid tumors. Its most typical clinical manifestations include cutaneous flushing, most often on the face, neck, and upper chest, diarrhea and abdominal pain [3]. Other manifestations may occasionally be encountered, including bronchial asthma, wheezing, or very rarely, skin manifestations [4].

Gastric carcinoid tumors are classified as those associated with chronic gastritis type A (CAG-A, or type I), those that concur with the Zollinger-Ellison syndrome (type II), and sporadic gastric tumors (type III). Type I gastric carcinoids account for 70 to 80 % of neuroendocrine gastric malignancies. They are associated with autoimmune atrophic gastritis, hypergastrinemia, and pernicious anemia [5]. Type I gastric carcinoids often measure less than 1 cm in diameter and are typically located in the body or fundus of the stomach [6, 7]. They are multicentric, polypoid, small, limited to the mucosa or submucosa, without angio-invasion, well-differentiated, and tend to display a benign behavior. They primarily affect women between the ages of 50 and 70 years and have a good prognosis, with a low rate of metastasis ranging between 2 and 5 % [8].

More than half of patients with CAG-A–associated carcinoids also have pernicious anemia. Pernicious anemia is associated with increased the risk of gastric carcinoid tumors, presumably due to prolonged achlorhydria. It results in parietal cell loss, compensatory hypergastrinemia, and argyrophilic cell hyperplasia [9].

Persistent achlorhydria produces G cell hyperplasia and more gastrin secretion, leading to hypergastrinemia [10]. Gastrin stimulates CCK-B/gastrin receptor (CCK2R) which further activates genes that encode HDC (histidine decarboxylase), VMAT2 (vesicular monoamine transporter molecule 2) and CgA (Chromogranin-A), but the signaling pathway leading to enterochromaffin cells hyperplasia remains unknown [11]. The widespread use of proton pump inhibitors can also induce gastric achlorhydria, thus contributing to hypergastrinemia.

Type II carcinoids present with hypergastrinemia and are associated with Zollinger-Ellison syndrome. In contrast to type I gastric carcinoids, the hypergastrinemia associated with type II gastric carcinoids is secondary to gastrin secreting G cell neoplasia rather than parietal cell loss. Type II gastric carcinoids are very uncommon; the majority have good prognosis. The last type of gastric carcinoid, type III, may be associated with atypical carcinoid syndrome [12, 13]. Atypical carcinoid syndrome is manifested by flushing, and tumors tend to be metastatic at their presentation, and thus have the worst prognosis [14]. Radical gastrectomy is the most common treatment for type III gastric carcinoids [15].

Here we report urticaria, angioedema and anemia as symptoms in an atypical presentation of type I gastric carcinoid. Our purpose is to highlight the relevance of cutaneous manifestations in the diagnosis and assessment of a patient with an underlying neuroendocrine malignancy. We also remark on the effectiveness of endoscopic ablation. And we encourage all medical practitioners to be aware that skin changes may provide an early clue for clinical management of gastric carcinoids.

## Case presentation

We report a case of a 50-year-old woman with atypical type I gastric carcinoid tumor. The patient presented with anemia, chronic urticaria and angioedema. The urticaria began one year prior to her evaluation by us, and was triggered by seafood. Other triggers included vitamin B12, desserts, and maize, as well as friction from shoes while running, and doing handicrafts. Lesions usually began in the first hour after the stimuli. Three months prior to developing urticaria, the patient was diagnosed with anemia. In the year since her urticaria began, the patient had lost 20 pounds without effort. She reported that her symptoms improved after taking the proton pump inhibitor (PPI) pantoprazole, but she only used the PPI sporadically. The reason for this response to PPI is not clear.

Initial symptoms of the urticaria were nausea accompanied by erythema, edema, pruritus and wheals. Wheals were larger than 5 mm, and were accompanied by erythema. They were associated with pain, burning and morning-time livedo reticularis. Wheals lasted more than 12 h, and were localized in palm, soles, armpits, feet, hands, buttocks, shoulders, trunk, legs and back. The patient also experienced generalized angioedema, which was triggered by seafood, corn, some desserts and ibuprofen, as well as blurred vision and sore throat. Urticaria Activity Score 7 (UAS 7) was 24, and the mean score on the CU-Q2oL quality of life measure was 3.61. Fecal exam, immunological tests (complement levels and immunoglobulins) and skin prick allergy tests for shrimp, crab, dust mite, and dog hair were all normal. There were no signs of vasculitis in the skin biopsy.

Laboratory tests showed diminished iron, ferritin, and vitamin B12 (patient had stopped taking B12, as it triggered the hives). The gastrin level was 286 pg/ml (reference value <100 pg/ml) and parietal cell antibody was 106.53 U/ml (reference value Negative: < or =20.0 U, Equivocal: 20.1-24.9 U, Positive: > or =25.0 U) [16]. RDW (Red Cell Distribution Width) was high, and anisocytosis was found. Microcytosis was also observed, perhaps due to iron deficiency, as well as megaloblasts.

**Fig. 1:**
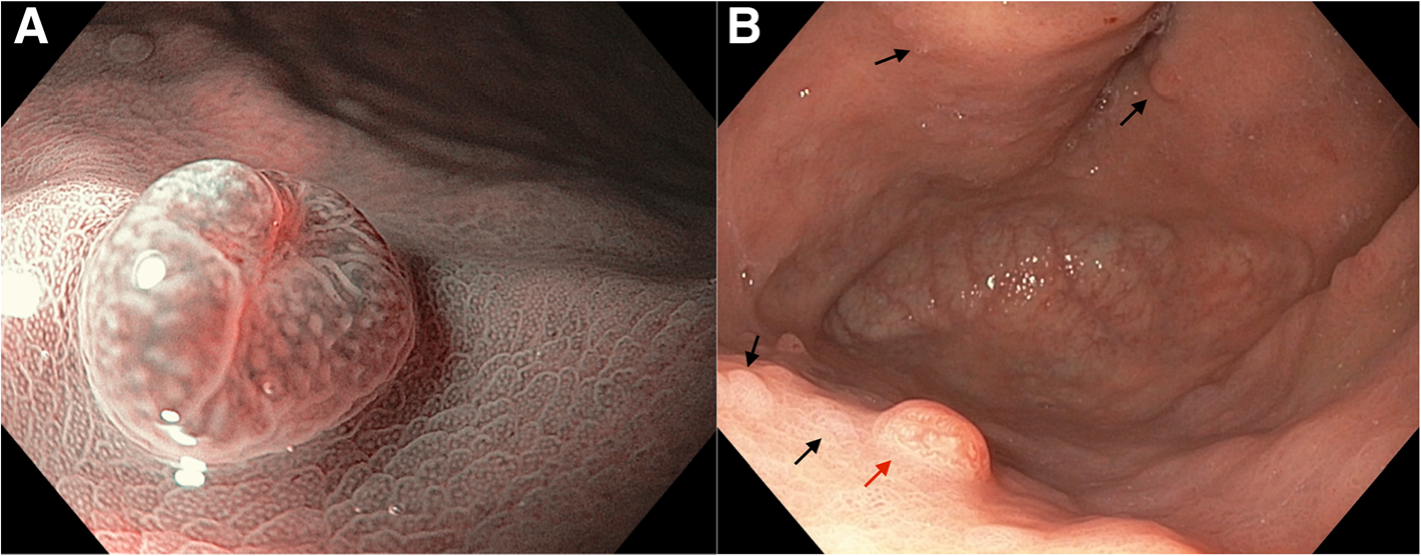
Examples of observed polypoids. **a** Sessile polypoid located in the greater curvature (magnification of **b**), 5 mm in diameter, depressed in its center and umbilicated, and alteration of glandular pattern (*red arrow* in **b**). **b** Black arrows indicate many sessile polypoids, with characteristics as described in **a**, located in the greater curvature in the body of stomach

Gastric endoscopy was performed and 15 sessile polyps with diameter of 5 mm were observed in the greater curvature of the stomach (three in the antrum and 12 in the body). Three in the antrum were extirpated (biopsy) and sent to pathology; the rest were subjected to plasma argon ablation (Fig. 1a-b). Also, biopsy of hyperplasia of mucosae located in the antrum of the greater curvature of the stomach was taken. No *H. pylori* bacilli were present, as determined by toluidine blue, but mild chronic gastritis with foveolar hyperplasia was identified.

This patient had four additional tumors in the small intestine, which were found upon examination via video capsule. The patient underwent endoscopic argon plasma therapy, which proved to be effective in resolution of urticaria. Quality of life greatly improved, with a mean CU-Q2oL score of 0 after treatment. Urticaria was successfully treated, with the UAS 7 score dropping to 0.

While endoscopic ablation has been associated with a high rate of recurrence, it is considered a safe procedure with 100 % survival, and has been demonstrated to be effective. We recommend further endoscopic follow-up to screen any possible recurrence.

Guidelines on follow up management of patients who have had endoscopic ablation of type I gastric carcinoid tumors have not yet been established. However, Yarzu et al. reported using omalizumab in postoperative care of a male patient with food allergy with pulmonary carcinoid tumor. In the post-surgery period the patient had recurrent laryngeal edema and urticaria attacks. Omalizumab treatment was prescribed because the patient was resistant to anti-histamines and steroids and was used for eight months in symptomatic therapy of recurrent laryngeal edema and urticaria attacks During the four years of follow up, no carcinoid tumor recurrence was noted [17].

## Conclusions

There are only a few reported cases of urticaria or angioedema associated with carcinoid tumors [4, 18]. The case reported here is particularly interesting because the tumors were located in the foregut. Foregut tumors generally do not secrete as much of the urticaria mediator kinin as do midgut tumors, and only secrete a small amount of the possible mediator serotonin [19]. However, foregut tumors may secrete 5-hydroxytryptophan (5-HTP), histamine or adrenocorticotropic hormone [2, 20]. Histamine release could lead to urticaria, which should respond to H1-antagonists [20]. Our patient was treated with H1 antagonists without response.

This patient presented with urticaria and angioedema as symptoms. Remarkably, urticaria was the reason for medical consultation. The patient had no diarrhea, and no signs or symptoms of bronchoconstriction or heart failure. This, then, would not fit the definition of a classical carcinoid syndrome [21]. The patient also presented with hypergastrinemia and anemia. This anemia can be attributed to several etiologies, since RDW was high, and anisocytosis, microcytosis and megaloblasts were observed. The patient presented chronic autoimmune gastritis, with high titers of anti-parietal cell antibodies, suggesting parietal cell loss as the etiology of G cell hyperplasia.

We strongly encourage all physicians who are looking for the etiology of any chronic urticaria, be they allergists, dermatologists, internists or primary care physicians, to consider the possibility of neuroendocrine malignancies and to screen these patients for elevated anti-parietal cell antibodies.

## Consent

Written informed consent was obtained from the patient for publication of this Case Report and any accompanying images. A copy of the written consent is available for review by the Editor-in-Chief of this journal.

## Abbreviations

5-HTP: 5-hydroxytryptophan
ACTH: adrenocorticotropic hormone
CAG-A: chronic gastritis type A
CU: chronic urticaria
CU-Q2oL: Chronic Urticaria Quality of Life Questionnaire
UAS 7: Urticaria Activity Score - 7
